# Consumers' Knowledge, Attitudes, and Practices Toward Medicine Price Transparency at Private Healthcare Setting in Malaysia

**DOI:** 10.3389/fpubh.2021.589734

**Published:** 2021-08-24

**Authors:** Nur Sufiza Ahmad, Ernieda Hatah, Mohamad Ridha Jalil, Mohd Makmor-Bakry

**Affiliations:** ^1^Faculty of Pharmacy, Universiti Kebangsaan Malaysia, Kuala Lumpur, Malaysia; ^2^Pharmaceutical Services Programme, Ministry of Health, Petaling Jaya, Malaysia

**Keywords:** price transparency, medicine price, consumer, knowledge, attitude, practice

## Abstract

**Background:** Medicine price transparency refers to the practice of making prices available to consumers for them to identify, compare, and select the medicine that provides the desired value. This study aimed to evaluate consumer knowledge, attitudes, and practices regarding Malaysia's medicine price transparency initiative, as well as factors that may influence related good consumer practices in private healthcare settings.

**Methods:** A cross-sectional, self-administered survey was conducted between May and July 2019 among consumers attending private healthcare institutions in Malaysia. The self-developed and validated survey consisted of four sections on the following: respondents' demographics, and 28 close-ended and graded Likert scale answer options on knowledge, attitudes, and practices toward medicine price transparency. Factors influencing good consumer practices toward the transparency initiative were modeled using binary logistic regression.

**Results:** A total of 679 respondents were part of the study. The mean age of respondents was 38 ± 13.3, with the majority (*n* = 420, 61.9%) being female. The respondents' mean score of knowledge and attitudes toward the price transparency initiative was 5.6 ± 1.5 of the total score of 8 and 31.9 ± 4.0 of the total score of 40, respectively. The respondents had the lowest score in the practice of price transparency, with a mean score of 31.5 ± 5.6 of the total score of 60. Male gender, Chinese ethnicity, high score on knowledge and attitudes, and high expenses on medicines influenced respondents' good practices of medicine price transparency.

**Conclusion:** Respondents had good knowledge and attitudes, but their usage and implementation of the medicine price transparency initiative was still inadequate. A number of factors influence this inadequacy, including gender, race, consumers' out-of-pocket spending on medication, and knowledge of and attitudes toward price transparency practices. Consumer-driven market price control would be impossible to achieve without the good consumer practices of medicine price transparency.

## Introduction

Price transparency in pharmaceuticals have been used in several countries as a strategy to help reduce expenditure on medicines. It can be defined as the practice of making prices available to consumers and/or to the government or the authority responsible for controlling or setting the market price of medicines. This is usually achieved through various mechanisms, such as the prices being published on the government/relevant website, displayed at the healthcare facilities, and printed on medicine labels or consumers' receipts and medical bills (1, 2). Australia, New Zealand, Lebanon, Oman, and Tunisia are examples of countries known to participate in medicine price transparency (3). They have accomplished this by publishing the prices on their government's website for the use of consumers. This transparency initiative helps consumers and the government to identify, compare, and choose medicines that offer the desired value (4). It creates awareness of price discrimination, which leads to informed choices and cost-saving by the users (5–9). It is likely to save the consumers' out-of-pocket spending by helping them with value-based purchasing and allowing them to exercise their right to price information before purchase (10, 11). Nonetheless, communication, education, and information about good medicine purchasing behavior are reported to have an impact on overall consumer behavior (12). Thawani et al. discovered that in India, after an information, education, and communication intervention, consumers' awareness of drug price variation, attitudes toward expensive and brand medicines, and behavior of comparing drug price information improved among 500 consumers (12). They concluded that consumers use medicines based on their knowledge, perceptions, and habits.

In Malaysia, the healthcare system is divided between public and private. The public healthcare system is offered to all Malaysian citizens at a low cost as it is highly subsidized by the government (13). The private healthcare system is funded through private insurance, employer benefits, and out-of-pocket payment. Although the public healthcare system is highly subsidized, patients may also choose to seek treatment in private healthcare settings such as retail pharmacies, private hospitals, and clinics (13). The government controls the price of medicines in the public healthcare system through direct negotiation and bulk purchasing. Nonetheless, in the free market, there is no price control for medicines supplied in private healthcare settings (14). It is estimated that 60% of pharmaceutical usage in private healthcare comes from consumers' out-of-pocket expenses (15).

Due to the lack of price control in private healthcare, the medicine prices are reported to be high and varied (14, 16–18). Based on a Ministry of Health (MOH) study over 2011–2015, the markup for generic and innovator medicine prices was between 31–402% (median 96%) and 24–86% (median 39%), respectively (14). The Malaysian government implemented the medicine price transparency initiative in 2011 as part of the National Medicine Policy (MNMP) to ensure consumer access to affordable medicines (19). The initiative includes the pharmaceutical industry's voluntary disclosure of medicine reference prices to the government and the public (20). Since the policy's launch in 2011, Malaysian consumers' knowledge, attitudes, and practices regarding medicine price transparency have remained unstudied. Although the National Survey on the Use of Medicines (NSUM) discovered that 68% of consumers believe that price label information is helpful in making an informed decision when purchasing medicines, it is not known whether this is actually practiced in real life (21). Other government initiatives aimed at increasing price transparency include strengthening the provision of itemized billing, which specifies the price of each item at all dispensing outlets, thus allowing for price comparison and reporting by the public (19). Despite the government's initiative to encourage consumers to practice medicine price transparency when purchasing medicine or receiving treatment in the private healthcare system, it is unclear whether Malaysian consumers have used the provision of itemized billing.

Therefore, the purpose of this study was to assess consumers' knowledge, attitudes, and practices regarding medicine price transparency initiatives, such as itemized billing and price comparison, as well as to investigate factors that may influence consumers good medicine purchase practices in Malaysia's private healthcare settings. The study also sought to shed light on whether it is possible to control medicine market prices in private healthcare through consumers' good purchasing practices.

## Materials and Methods

### Study Design and Sampling

This study was conducted as a cross-sectional survey among the public between May and July 2019. Individuals aged 18 years and above, Malaysian citizens, and those with experience in out-of-pocket purchases from private healthcare facilities in Malaysia were invited to participate in the study. Using the Raosoft sample size calculator for a survey study and a confidence interval of 95%, a margin of error of 5%, and a Malaysian adult population of 22 million (22), an estimated sample size of 385 was required. Respondents were excluded from the study if they did not complete 80% of the questionnaire, understand English or Malay, or provide informed consent. Using convenient sampling, the self-administrated survey was distributed face-to-face and online using a Google form shared through social media channels, such as Facebook and WhatsApp. The face-to-face survey was distributed across the country in public places in urban and rural areas, such as shopping malls, community pharmacies, clinics, and community halls. Participation in the study was entirely voluntary and without remuneration. Respondents who agreed to participate were asked to sign the informed consent form or click the agreement button before answering the survey questions.

### Survey Instrument

The questionnaire was developed through a literature review of reports and documents related to the medicine price transparency initiative (21, 23, 24) and inputs from domain experts. It consisted of four sections: (a) respondents' demographics and characteristics, (b) knowledge−8 items, (c) attitudes−8 items, and (d) practices related to consumer rights on medicine purchasing−12 items. Section A gathered respondents' information on age, gender (male or female), race (Malay, Chinese, Indian, or other races), highest level of education, occupation, monthly income, area of residence (urban or rural), medical coverage (insurance health policy or employer medical coverage), healthcare condition, amount of money spent on medicine, facilities where medications were usually obtained, and method used to access medicine price information. In the remaining sections, respondents were asked about their knowledge, attitudes, and practices regarding their rights when receiving medications, such as price information, itemized billing, and filing a complaint if any problem. They were provided with “yes,” “no,” or “not sure” answer options in Section B, five Likert-scale answer options ranging from “strongly agree” to “strongly disagree” in Section C, and “never” to “always” in Section D. The score for each section was calculated using the sum of the scores for a correct answer or a score between one to five for the statement with negative attitudes or “never practice,” and most positive attitudes and “always” practice. Reverse-scoring was given for all negative statements accordingly. Using Bloom's cut-off point, respondents' practice score on medicine price transparency was categorized as good if they had a sum score of 60% and above, which is a combination of a high and moderate score, and poor for a score <60% (25).

### Data Analysis

The content validity of the questionnaires was evaluated by two academicians, two MOH pharmacy officers, and two independents reviewers who were experts in medicine pricing and/or consumer surveys. The content validity index (CVI) and the average scale-level CVI (S-CVI/Ave) was conducted to measure the relevancy and clarity of the statements and its proportion relevance judged by all expert (26). The result of the item-CVI (I-CVI) for statements in each section was between 0.83 and 1, with S-CVI/Ave of 0.93, 0.96, and 0.92 for the knowledge, attitudes, and practices sections, respectively. The survey was initially prepared in English and translated to Malay by two independent translators using backward and forward translation. To ensure clarity and reliability, the questionnaires were pilot tested with 30 members of the public. The Cronbach's alpha coefficient (r) for Sections B, C, and D were found to be reliable at 0.70, 0.76, and 0.75, respectively (27).

The data were analyzed using the IBM SPSS software Version 24 for descriptive and inferential analyses. Factors likely to influence respondents' good practices of rights when purchasing medicines, with a score of >60%, were modeled using binary logistic regression with a stepwise-backward approach. The variables tested were respondents' demographics and characteristics, as well as their knowledge and attitudes score on medicine purchasing behavior, with a *p* < 0.05 considered significant. Prior to the binary logistic regression, a univariate analysis was performed to determine which variables would be included in the final model analysis. Variables were included if the adjusted odds ratio had *p* < 0.25 (28).

## Results

A total of 679 responses were received for the study, with 406 completed face-to-face and 273 completed online. Ten respondents were excluded from the study because their responses were <80% complete. As a result, a final 679 responses were included in the analysis. A summary of the respondents' demographics and characteristics is presented in Table 1. The mean age ± standard deviation of the respondents was 38 ± 13.3, with a majority (*n* = 420, 61.9%) being female and of Malay ethnicity (*n* = 459, 67.6%). A total of 422 (65.1%) had a diploma or bachelor's degree as their highest education level. The majority (*n* = 465, 68.5%) worked and had a monthly income of less than RM3000 (*n* = 382, 56.2%). More than half (*n* = 421, 60.0%) had private insurance or employer benefit coverage and were healthy. Only 135 respondents (19.9%) had serious health problems, such as cardiovascular disease, diabetes mellitus, hypercholesterolemia, asthma, gastroenteritis, and arthritis. A total of 353 (52.0%) respondents spent less than RM100 in a month for medicine, while a majority (*n* = 414, 61.0%) reported checking medicine price information before purchasing a medicine, with most practicing comparing the printed price at various healthcare facilities (*n* = 338, 49.8%).

**Table 1:** Respondents' demographics and characteristics.

**Variables**	**Descriptions**	***N* (%)/Mean (s.d)**
Gender	Male	259 (38.1)
	Female	420 (61.9)
Age		38 (13)
Race	Malay	459 (67.6)
	Chinese	120 (17.7)
	Indian	62 (9.1)
	Others	38 (5.6)
Highest education level	College/University	422 (65.1)
	Upper middle school	153 (22.5)
	Lower school	15 (2.2)
Occupation	Employed	465 (68.5)
	Unemployed/housewife	66 (9.7)
	Student	122 (18.0)
	Pensioner	26 (3.8)
Monthly income (RM)	<1,000	206 (30.3)
	1,000–3,000	176 (25.9)
	3,001–6,000	181 (26.7)
	>6,000	116 (17.1)
Living area	Urban	565 (83.2)
	Rural	114 (16.8)
Private insurance health coverage	No coverage	258 (38.0)
	Private insurance/Employer benefit	421 (62.0)
Health status	Has health problem	135 (19.9)
	Do not has heath problem	544 (80.1)
Medicine expenditure per year (RM)	<100	353 (52.0)
	100–500	256 (37.7)
	500–1,000	44 (6.5)
	>1,000	26 (3.8)

The respondents' mean score of knowledge of the medicine price transparency initiative was 5.6 ± 1.5 from the maximum score of 8. A majority of the respondents knew they had the right to get their medication anywhere they preferred (*n* = 517 76.1%), were aware that the price of the same medicine could be different at different healthcare facilities (*n* = 550, 81.0%), and that they were entitled to receive an itemized bill from their health practitioners (*n* = 629, 92.5%). However, only 318 (46.8%) of the respondents knew about the medicine price guide on the Pharmaceutical Service Programme (PSP) website. A total of 258 (38.0%) were not sure they could make a complaint to the MOH about overcharging on medical and medicine bills. Table 2 presents a summary of the respondents' scores on knowledge of the medicine price transparency initiative in Malaysia.

**Table 2:** Respondents' knowledge of medicine price transparency in private healthcare setting in Malaysia.

**No**	**Statement**	**Yes**	**No**	**Not sure**
		***N* (%)**	***N* (%)**	***N* (%)**
1.	I have the right to get my medication anywhere at my preference, not necessarily from the doctor who is treating me.	517 (76.1)	103(15.2)	59 (8.7)
2.	I can request a change for a cheaper medication from my doctor/ pharmacist.	366 (53.9)	169 (24.9)	144 (21.2)
3.	Every patient is entitled to get a itemized bill from health practitioners (doctors or pharmacists).	629 (92.5)	11 (1.6)	39 (5.7)
4.	It is sufficient if the itemized bill lists only the total costs of the treatment and medications.	195 (28.7)	368 (54.2)	116 (17.1)
5.	I have the right to know the estimated charges for my treatment.	566 (83.4)	37 (5.4)	76 (11.2)
6.	I can check the medication price guide at the Pharmaceutical Service Programme, Ministry of Health website.	318 (46.8)	42 (6.2)	319 (47.0)
7.	The price of the medication at private hospitals, clinics and retail pharmacies may differ regardless of the same brand.	550 (81.0)	22 (3.2)	107 (15.8)
8.	Complaints about overcharging medical and medicine bill can be lodged with the Ministry of Health Malaysia.	398 (58.6)	23 (3.4)	258 (38.0)

The mean score on respondents' attitudes toward medicine price transparency in Malaysia was 31.9 ± 4.0 of the total score of 40. A majority of them (*n* = 539, 79.4%) “strongly agreed” and “agreed” about the need and importance of being aware of the medicine cost before purchase for making an informed choice (*n* = 598, 88.1%). A total of 604 respondents (88.9%) “strongly agreed” or “agreed” that price comparison helped them get the best price for their medicines. There were 571 (84%) respondents who “disagreed” with the need for requesting itemized bills, yet the majority (*n* = 623, 92.8%) “strongly agreed” and “agreed” that an itemized bill should include the price of each medicine rather than the total cost. A total of 211 (31.1%) respondents were “neutral” about paying high prices for medicines in private healthcare settings, while 155 (22.8%) “agreed” or “strongly agreed” that they did not mind paying higher prices for medicines in a private clinic or hospital. Table 3 presents a summary of the attitudes score of the respondents.

**Table 3:** Respondents' attitudes on medicine price transparency practice in private healthcare setting in Malaysia.

**No**	**Statement**	**Strongly agree**	**Agree**	**Neutral**	**Disagree**	**Strongly disagree**
		***N* (%)**	***N* (%)**	***N* (%)**	***N* (%)**	***N* (%)**
1.	A patient should know the costs of treatment beforehand.	273 (40.2)	266 (39.2)	101 (14.9)	27 (4.0)	12 (1.8)
2.	Price comparison before purchasing helps consumers to get the best price for their medication.	337 (49.6)	267 (39.3)	57 (8.4)	10 (1.5)	8 (1.2)
3.	I need not be concerned about the medication price if it is covered by my insurance company or my employer.	41 (6.0)	111 (16.3)	129 (19)	250 (36.8)	148 (21.8)
4.	I do not mind paying high price of medicine in a private clinic or hospital.	32 (4.7)	123 (18.1)	211 (31.1)	194 (28.6)	119 (17.5)
5.	It is not fair to set a high medicine price to those with higher income.	203 (29.9)	216 (31.8)	153 (22.5)	73 (10.8)	34 (5.0)
6.	The itemized bill should list out the price of each medication prescribed rather than just the total cost in a bill.	351 (51.7)	272 (40.1)	45 (6.6)	5 (0.7)	6 (0.9)
7.	It is not important to ask for itemized billing.	13 (1.9)	29 (4.3)	66 (9.7)	276 (40.6)	295 (43.4)
8.	It is important to know the medication charge to allow me to make a better choice.	292 (43)	306 (45.1)	65 (9.6)	10 (1.5)	6 (0.9)

Next, the respondents' mean score on practices related to medicine price transparency was 31.5 ± 5.6 of the total score of 60. A total of 125 respondents (18.4%) “never” asked the estimated price of medicines before receiving treatment, while 420 (61.81%) “always” and “very often” complied with the medicine choice made by their doctor, regardless of price. Only a small number of respondents (*n* = 108, 15.9%) “always” asked for an itemized bill. More than half “never” negotiated nor asked for a price discount when purchasing medicines at a retail pharmacy (*n* = 376, 55.4%) and private health clinics and hospitals (*n* = 471, 69.4%). Only 64 (9.4%) respondents “always” asked their healthcare provider for an explanation of speculative charges on their bill. Table 4 presents a summary of respondents' practices score on the price transparency initiative.

**Table 4:** Respondents' practice on medicine price transparency practice in private healthcare setting in Malaysia.

**No**	**Statement**	**Always**	**Very often**	**Sometimes**	**Rarely**	**Never**
		***N* (%)**	***N* (%)**	***N* (%)**	***N* (%)**	***N* (%)**
1.	I ask the estimated price of medication before getting any treatment.	104 (15.3)	122 (18.0)	204 (30.0)	124 (18.3)	125 (18.4)
2.	I check for the price of medications before purchasing at retail pharmacy.	144 (21.2)	200 (29.5)	145 (21.4)	101 (14.9)	89 (13.1)
3.	I compare the prices at different pharmacies prior deciding to purchase the medications.	97 (14.3)	148 (21.8)	183 (27)	136 (20)	115 (16.9)
4.	I ask for a cheaper brand of medication when I cannot afford the recommended medication price.	55 (8.1)	127 (18.7)	167 (24.6)	145 (21.4)	185 (27.2)
5.	I comply with the doctor's choice of medication regardless of the price.	134 (19.7)	286 (42.1)	172 (25.3)	60 (8.8)	27 (4.0)
6.	I ask for the itemized bill every time I seek treatment from any private clinic or hospital.	108 (15.9)	156 (23)	135 (19.9)	133 (19.6)	147 (21.6)
7.	Besides the price, I take into consideration the quality and effectiveness when purchasing medications.	261 (38.4)	256 (37.7)	86 (12.7)	40 (5.9)	36 (5.3)
8.	I ask for discount/negotiate the price of my medication at retail pharmacy.	32 (4.7)	45 (6.6)	82 (12.1)	144 (21.2)	376 (55.4)
9.	I ask for discount/negotiate the price of my medication at private clinic or hospital.	15 (2.2)	26 (3.8)	40 (5.9)	127 (18.7)	471 (69.4)
10.	I pay for my medication even if I feel that the price unreasonable.	96 (14.1)	229 (33.7)	186 (27.4)	71 (10.5)	97 (14.3)
11.	I ask for an explanation from my healthcare provider regarding any speculative charge in my bill.	64 (9.4)	172 (25.3)	140 (20.6)	115 (16.9)	188 (27.7)
12.	I complain to the authority if I feel that I am billed unreasonably.	15 (2.2)	52 (7.7)	52 (7.7)	97 (14.3)	463 (68.2)

The respondents' practices scores were then classified as good or poor using a percentage score that is the sum of the scores divided by the total score multiplied by 100. Only 220 (32.4%) had good practices, with a score of ≥60%, and the rest (*n* = 459, 67.6%) had poor practices in medicine price transparency, with a score of <60%. Gender, race, annual spending on medicines, knowledge, and attitudes scores were found to have a significant influence on respondents' good practices in price transparency [χ${}_{\left(11,\text{N}=679\right)}^{2}$ = 75.56, *p* < 0.001]. Male respondents were 1.78 times more likely than female respondents to apply good practices in medicine price transparency (AOR [95% CI] = 1.78 [1.26, 2.56], *p* < 0.001). Chinese (AOR [95% CI] = 1.96 [1.26, 3.04], *p* = 0.003) and other races (AOR [95% CI] = 2.29 [1.12, 4.67] *p* = 0.023) were also more likely to apply good practices than the Malays. The study also discovered that respondents who spent more than RM1000 on their medicines were 3.13 times more likely to practice good medicine price transparency than those who spent less than RM100 per month (AOR [95% CI] = 3.13 [1.33, 7.36], *p* = 0.009). Furthermore, a 1% increase in the knowledge and attitudes scores increased the likelihood of respondents engaging in good practices by 41% (AOR [95% CI] = 1.41 [1.24, 1.61], *p* < 0.001) and 6% (AOR [95% CI] = 1.06 [1.04, 1.11], *p* < 0.001), respectively. The final model on factors that influence respondents' good practices on medicine price transparency is presented in Table 5.

**Table 5:** Factors that may influence respondents' good practice toward medicine price transparency at the private healthcare setting.

**Variables**	**Poor practice *n* = 459 *n* (%)^a^**	**Good practice *n* = 220 *n* (%)^a^**	**Univariate analysis (** ***n*** **=** **679)**			**Multivariate analysis (** ***n*** **=** **679)**		
			**Crude OR**	**Wald's χ^**2**^**	***P*-value**	**Adj. OR**	**Wald's χ^**2**^**	***P*-value**
			**(95% CI)**	**(*df*)**		**(95% CI)**	**(*df*)**	
**Gender**								
Male	157 (60.6)	102 (39.4)	1.00	9.24 (1)	0.020	1.00	10.48 (1)	<0.001
Female	302 (71.9)	118 (28.1)	0.60 (0.43, 0.83)			0.56 (0.39,0.79)		
**Race**								
Malay	329(71.7)	130 (28.3)	1.00	11.77 (3)	0.008	1.00	12.44 (3)	0.006^*^
Chinese	68 (56.7)	52 (43.3)	1.94 (1.28, 2.93)		0.002	1.96 (1.26, 3.04)		0.003^*^
Indian	40 (64.5)	22 (35.5)	1.39 (0.79, 2.43)		0.246	1.22 (0.67, 2.22)		0.510
Others	22 (57.9)	16 (42.1)	1.84 (0.94, 3.62)		0.077	2.29 (1.12, 4.67)		0.023^*^
**Highest education**								NS
College/University	349 (68.3)	162 (31.7)	1.00	1.53 (2)	0.466			
Upper middle school	102 (66.7)	51 (33.3)	1.08 (0.73, 1.58)		0.705			
Lower school	8 (53.3)	7 (46.7)	1.89 (0.67, 5.29)		0.228			
**Occupation**								NS
Employed	311 (66.9)	154 (33.1)	1.00	7.30 (3)	0.063			
Unemployed/housewife	49 (74.2)	17 (25.8)	0.70 (0.39, 1.26)		0.233			
Student	87 (71.3)	35 (28.7)	0.81 (0.53, 1.26)		0.352			
Pensioner	12 (46.2)	14 (53.8)	2.36 (1.06, 5.22)		0.035			
**Monthly income (RM)**								NS
No income and <1,000	143 (69.4)	63 (30.6)	1.00	1.34 (4)	0.855			
1,000–3,000	119 (67.8)	57 (32.4)	0.92 (0.53, 1.62)		0.778			
3,001–6,000	121 (66.9)	60 (33.1)	0.96 (0.55,1.67)		0.872			
>6,000	76 (65.5)	40 (34.5)	1.01 (0.56, 1.85)		0.965			
**Location of living**								
Urban	382 (67.6)	183 (32.4)	1.00	0.00 (1)	0.989			NS
Rural	77 (67.5)	37 (32.5)	1.0 (0.65, 1.54)					
**Health coverage**								NS
No coverage	185 (71.7)	73 (28.3)	1.00	3.19 (1)	0.074			
Private Insurances/Employer benefit	274 (65.1)	147 (34.9)	1.36 (0.97,1.90)		<0.001			
**Disease status**								NS
Has health problem	88 (65.2)	47 (34.8)	1.00	0.45 (1)	0.503			
No health problem	371 (68.2)	173 (31.8)	0.87 (0.57, 1.30)					
**Medicine expenditure per year (RM)**								
<100	251(71.1)	102 (28.9)	1.00	12.37 (3)	0.006	1.00	7.89 (3)	0.048^*^
100–500	172 (67.2)	84 (32.8)	1.20 (0.85, 1.70)		0.300	1.17 (0.81, 1.69)		0.398
500–1,000	26 (59.1)	18 (40.9)	1.70 (0.89, 3.24)		0.105	1.56 (0.79, 3.07)		0.201
>1,000	10 (38.5)	16 (61.5)	3.94 (1.73, 8.97)		0.001	3.13 (1.33, 7.36)		0.009^*^
Age	37.5^b^ (12.5)^c^	39.9^b^ (14.4)^c^	1.02 (1.00, 1.03)	5.42 (1)	0.020			NS
Knowledge score	5.4^b^> (1.5)^c^	6.1^b^ (1.3)^c^	1.46 (1.29, 1.66)	34.92 (1)	<0.001	1.41 (1.24, 1.61)	26.51 (1)	<0.001
Attitude score	31.6^b^ (4.1)^c^	32.7^b^ (3.5)^c^	1.08 (1.03, 1.13)	12.17 (1)	<0.001	1.06 (1.01, 1.11)	6.52 (1)	<0.011

* *Significant p < 0.05*.

a *The percentage is reported by column, adding up to 100% based on available information*.

b *Mean*.

c *SD*.

*COR, crude odds ratio; AOR, adjusted odd ratio; CI, confidence interval; NS, non-significant*.

## Discussion

This study provides an overview of Malaysian consumers' knowledge, attitudes, and practices regarding medicine price transparency in private healthcare settings. It found that the majority of respondents scored higher in knowledge of and attitudes toward the medicine price transparency initiative than in practice. One related area where respondents were found to have a lack of knowledge concerns the medicine price guide on the PSP website (29). This could be because the website was relatively less known to the public, and/or respondents preferred to physically check prices at the facilities rather than online. Therefore, increasing medicine price transparency physically in the healthcare settings, as practiced in stores or clinics in the Philippines and hospitals in Thailand, will be a good alternative to increasing consumers' medicine price transparency practices (1, 2). Since 2019, private hospitals in Thailand have been required to display their medicine prices on their advertisement board, website, or via QR scan codes (2). If such a practice is implemented in Malaysia, consumers would be able to verify the medicine prices using scanned QR codes from the healthcare facilities of their choice (2). Checking drug prices before purchasing them would protect consumers from being overcharged and provide them with an opportunity to discuss their concerns about drug costs with their doctors (30). Furthermore, it can increase consumer confidence in negotiating drug pricing, allowing them to obtain their medication at reasonable prices and continue their treatment affordably. More information, education, and communication interventions are also required to improve Malaysian consumers' behavior when purchasing medicines or receiving treatment in private healthcare settings.

In this study, a majority of the respondents (91.8%) agreed on the importance of obtaining itemized bills following their treatment. Nevertheless, this did not translate into practice as only 15.9% of the respondents “always” practiced obtaining itemized bills. This could be because, first, the Malaysian Private Healthcare Facilities and Services Regulation specifies that itemized bills are required in private healthcare settings only if the patient requests for it (31, 32). As a result, providing itemized bills for patients is not a usual practice, particularly in primary healthcare settings such as clinics and pharmacies. Second, because itemized billing is a non-voluntary practice, patients may perceive receiving a non-itemized bill as good consumer behavior and would not ask for an itemized bill. Finally, experience with compliance with non-itemized bills may be a reason why patients do not request them. According to a study on private hospital billing in Malaysia, some hospitals did not follow the suggestion for itemized billing, for example, when the treatment costs such as for medicines were presented as a lumpsum amount or in combination with other item costs such as consumables (33). To ensure compliance with the itemized billing regulation, stricter penalties and monitoring are necessary. In addition, instead of issuing itemized bills only on patient request, the government may consider making them mandatory, leading to a better selection of medicines by patients and prevention of overcharging.

The respondents were also found to be more likely to comply with a doctor's choice of medicine, regardless of price, and less likely to negotiate on the medicine price or ask for a discount. This could be because consumers are more concerned about receiving effective treatment regardless of cost, as reported in previous studies (30, 34), or because they really trust their healthcare providers (35). The study by Schafheutle et al. in England reported that a majority of their patients rarely and reluctantly discussed medicine prices and their affordability with their general practitioners (30). This is often because they felt reluctant to discuss due to the short consultation time, or, as stated, did not want to jeopardize the relationship they had with their doctor. Similarly, Fraeyman et al.'s study of patients with chronic diseases in Belgium revealed that <4% of the participants discussed medicine price issues with their doctors or pharmacists (36). Thus, for healthcare providers to include and initiate discussions about medicine prices and affordability with their patients should be encouraged.

In total, 28% of the respondents of this study stated that they “rarely” or “never check” their medicine price before purchasing. This is similar to the 2013 study by Baber and Ibrahim on consumer attitudes on affordability of medicines in Malaysia, where 37% of the respondents did not check the medicine price before purchasing them (23). This demonstrates that, even after many years, consumer behavior in purchasing medicine has not changed and must be urgently improved for their welfare.

In this study, male gender, Chinese ethnicity, high knowledge and attitudes scores, and high medicine expenditure cost were found to influence good consumer practices regarding medicine price transparency. In line with previous NSUM findings, all demographic variables, including race and ethnicity, were significantly associated with medicine price label checking and purchasing behavior (21). Because knowledge and attitudes can influence good consumer behavior in terms of medicine price transparency, it is critical to educate and communicate with consumers on a regular basis to raise their awareness and practice of price transparency. Interventions such as printed handouts, press releases, and interactive discussion sessions on medicine pricing have been found to be effective in changing consumer behavior in India, such as comparing prices before purchasing and being concerned about aspects of medicine use (12). Furthermore, respondents who had a high out-of-pocket expenditure on medicines were more likely to use the price transparency initiative to reduce their treatment costs (37).

There are some limitations to this study. First, patients who can afford to pay for the service or who have health insurance or employer benefit coverage are more likely to use private healthcare services in Malaysia. As a result, this study may have excluded people who could not afford treatment in private healthcare settings. Second, because socioeconomic status influences treatment choice in Malaysia, with the wealthier seeking care in private healthcare settings (38), the study's findings may be influenced by the differences in consumer socioeconomic status and payment schemes. Nonetheless, respondents from various socioeconomic backgrounds were included in this study to ensure that the findings were generalizable to the public on average. Third, the cross-sectional study design represents the findings at one point in time and does not reflect future findings of consumers' knowledge, attitudes, and practices on medicine price transparency. Last, the nature of the survey required patients to recollect their purchasing behavior practices, which may be open to recall bias, which is common to such survey study designs (39).

## Conclusion

In summary, despite good knowledge and attitudes scores among the consumers, the practice of attaining medicine price transparency is still unsatisfactory and inadequate in Malaysia. A number of influencing factors were found, including gender, race, consumers' out-of-pocket spending on medicines, and knowledge and attitudes scores in price transparency practices. Consumer-driven market price control would be impossible to achieve without good consumer practices related to price transparency, such as asking for itemized bills, and checking, comparing, and negotiating the price of medicines. Aside from educating and raise consumer awareness about the importance of medicine price transparency, government intervention such as compulsory itemized bills and increase medicine price transparency physically in the healthcare settings are required.

## Data Availability Statement

The raw data supporting the conclusions of this article will be made available by the authors, without undue reservation.

## Ethics Statement

The studies involving human participants were reviewed and approved by Human Research Ethics Committee, Universiti Kebangsaan Malaysia (UKM PPI/111/8/JEP-2019-060). The patients/participants provided their written informed consent to participate in this study.

## Author Contributions

NA conceptualized and designed, conducted data collection, analyses, and drafted the initial manuscript. EH and MM-B conceptualized and designed the study, interpretation of data and reviewed, and revised the manuscript. MJ conceptualized, conducted data collection, and revised the manuscript. All contributors approved the final manuscript as submitted and had complete access to the study data that support the publication.

## Conflict of Interest

The authors declare that the research was conducted in the absence of any commercial or financial relationships that could be construed as a potential conflict of interest.

## Publisher's Note

All claims expressed in this article are solely those of the authors and do not necessarily represent those of their affiliated organizations, or those of the publisher, the editors and the reviewers. Any product that may be evaluated in this article, or claim that may be made by its manufacturer, is not guaranteed or endorsed by the publisher.
